# Molecular Characterization of Cefoxitin-Resistant Coagulase-Negative Staphylococci From Frequently Touched Surfaces of Hospital and Urban-Built Environments of Central India

**DOI:** 10.1155/cjid/5766823

**Published:** 2025-02-05

**Authors:** Anushri Keshri, Dilip Govardhan Gore, Indu Singh, Divakar Sharma, Varaprasad Kolla

**Affiliations:** ^1^Department of Biotechnology, Amity Institute of Biotechnology, Amity University Chhattisgarh, Raipur, Chhattisgarh 493225, India; ^2^Department of Biotechnology, Sai Biosystems Private Limited, Nagpur, Maharashtra 440009, India; ^3^Department of Pharmacy, School of Pharmacy, Graphic Era Hill University, Dehradun 248002, India; ^4^Department of Biotechnology, Graphic Era Deemed to be University, Dehradun 248002, India

**Keywords:** coagulase-negative staphylococci (CoNS), *mecA* gene, multidrug-resistant (MDR), SNP analysis, surveillance program

## Abstract

Coagulase-negative staphylococci (CoNS) are the major pathogen (hospital as well as environmental) and their emerging multidrug-resistant (MDR) strains complicate the treatment process. In this study, we investigated the prevalence and antibiotic resistance of CoNS on frequently touched surfaces in hospital and urban built environments (UBEs) in Vidarbha, Maharashtra, India. A total of 200 isolates screened for *Staphylococcus* species and 55 methicillin-resistant staphylococci isolates were identified, and among them, 19 were classified as cefoxitin-resistant CoNS. These 19 cefoxitin-resistant CoNS isolates were tested for the presence of the *mecA* gene by conventional PCR and only nine (47.36%) were found to be *mecA-*positive. *mecA-*positive strains were tested to check MIC for various antibiotics and three marker gene characteristics, namely, ß-lactamase, cefoxitin screen, and inducible clindamycin resistance via the VITEK 2 system. These strains were 100% resistant to benzylpenicillin and oxacillin, and approximately 50% were resistant to vancomycin. Amplified *mecA* gene fragments were sequenced, and SNP analysis was performed alongside a standard sequence from *Staphylococcus aureus* (Acc no. NG_047938.1). In total, among the 466 nucleotides, 386 sequences were found to be invariable, and 80 polymorphic variables were identified (46 singleton variable sites and 34 parsimony information sites). The spread of antibiotic resistance is very common in both UBEs and hospital environments; thus, our study concluded that a surveillance program is recommended for the Vidarbha region for the assessment of co-occurring CoNS and better infection control of the environment for future reduction in contact infection.

## 1. Introduction

Coagulase-negative staphylococci (CoNS) are generally recognized as less pathogenic or nonpathogenic than *Staphylococcus aureus*. Among the heterogeneous groups of CoNS, *Staphylococcus epidermidis* and *Staphylococcus haemolyticus* are recognized as major nosocomial pathogens. However, it is reported that they have low virulence factors but still have a large proportion of methicillin-resistant (MR) strains, which makes them less susceptible to glycopeptides and other therapeutics [1]. Nowadays, CoNS were found to be related to nosocomial infection. CoNS were speculated for the presence of virulence factors through molecular typing as well as whole-genome sequencing/WGS [2]. According to scientific experts, CoNS has a substantial impact on morbidity and socioeconomic costs. Becker et al. reported that an increasing number of CoNS cases are linked to immunocompromised patients, demographic environment, and hospital-related factors, which is the rationale of the present study [3]. According to Koch's postulates, CoNS is considered to be low in pathogenicity, but due to the presence of virulence factors and several antibiotic-resistant genes, CoNS is considered highly pathogenic that impacts human health [4].

*Staphylococci* are the nosocomial pathogens that cause hospital-associated infections (HAIs) and the spread of MR *staphylococci* in hospitals was considered a massive threat to patients and hospital staff. However, less has been explored related to the dissemination spectrum of staphylococci, especially, when possible, through frequently touched surfaces in hospital environments [5]. Among the bacterial populations on frequently touched surfaces, CoNS are the most prevalent than *S. aureus* and *P. aeruginosa*, though it is important to investigate the pathogenicity of these less explored strains [6]. As reported, frequently touched surfaces in hospitals can harbor both potentially pathogenic and nonpathogenic bacterial species. The reason for pathogenic transmission is due to the incorrect execution of hand hygiene by healthcare workers/hospital staff, patients, and their caretakers, via touched surfaces and materials [7]. Investigation of CoNS from frequently touched surfaces of hospital settings and urban built environments (UBEs) is crucial because the persistence of these bacteria on such surfaces poses a higher risk of spreading multidrug-resistant (MDR) strains such as MR CoNS (MRCoNS) which not only complicates the management of infections but also inflate healthcare costs. The microbial burden in UBEs, which was less studied, can significantly influence community-acquired infections. The presence of MDR–CoNS in such environments raises concerns about their potential to serve as reservoirs of MDR genes, which can be transferred to other pathogens, amplifying the antimicrobial resistance problem. Monitoring the MDR bacteria and antibiotic-resistant pattern could be employed to identify risks, improve hygiene practices, control the spread of resistant pathogens, and protect public health in diverse environments [5–8].

Mechanistically, antibiotic resistance is due to the presence and expression of antibiotic-resistant genes in the microorganism, which is acquired in the genome and further, inherited from generation to generation. The virulence and resistance vary due to the presence of prevalent single nucleotide polymorphisms (SNPs) among these genes [9]. By whole-genome sequencing or targeting genomics of CoNS, many genes were detected and linked with drug resistance and mutations [10]. In recent eras, genome blast phylogeny, gene-specific phylogeny, and SNP analysis were used together to diversify the population based on sequence information to correlate the virulence, spread, mortality, mutation, and other factors [11]. In the present study, the prevalence of MRCoNS was confirmed in UBEs and hospital-touched surface environments. Bacterial isolates confirmed for MR were identified at the species level and subsequently subjected to *mecA* gene sequencing. Later, the *mecA* gene was analyzed for SNP and phylogeny. For infection control strategy, the minimum inhibitory concentration (MIC) of MRCoNS was performed for a range of antibiotics to elucidate the resistance profile which ultimately led to managing the MRCoNS in the Vidarbha region of Maharashtra, India.

## 2. Materials and Methods

Nagpur district of the Vidarbha region is the most developed city and is considered as a promising healthcare hub, and patients visit hospitals from nearby areas and across the Maharashtra state. Therefore, Vidarbha is an important site to investigate human pathogens such as *Staphylococcus* species with special attention to CoNS for drug resistance. The sampling from the touched surfaces of UBEs and hospital settings may provide us with an overview of the microbial prevalence and resistance profile.

### 2.1. Research Approval

Research approval was granted by the Institutional Research Committee of the University, India, and the approval number is AUC/IEC/AIB/23/112.

### 2.2. Sample Collection From Contaminated Places

In the present study, 200 *staphylococcus* isolates were isolated from the collected samples of various frequently touched surfaces in UBEs and hospital settings. After swapping, the sterile swabs were immediately transferred to 0.9% sterile saline and processed in the laboratory within 2–4 h.

### 2.3. Isolation and Identification of *Staphylococcus* Species or CoNS

Swabs from the touched surfaces (UBEs and hospital settings) were immediately spread on selective mannitol salt agar (MSA) media. The plates were incubated at 37°C for 24–48 h for colony formation, after which colonies were maintained on Mueller–Hinton Agar (MHA) plates only. Gram staining was performed, and coagulase-negative features were checked. In biochemical assay, catalase activity was tested for confirmation of the CoNS. Further identification, reconfirmation, or validation was performed at the species level by VITEK 2 as per the manufacturer's protocol.

### 2.4. Antibiotics Susceptibility Testing (AST) of CoNS by Disc Diffusion Method

After the CoNS confirmation, they were further screened for antibiotic susceptibility by the disc diffusion method on MHA and recording their drug resistance to various drugs such as ampicillin (10 μg), methicillin (10 μg), oxacillin (1 μg), amoxicillin (10 μg), cefoxitin (30 μg), fusidic acid (10 μg), cefepime (30 μg), mupirocin (20 μg), gentamicin (10 μg), erythromycin (15 μg), penicillin G (1 unit), and piperacillin (100 μg) (HiMedia, India). The observed resistance was analyzed as per the Clinical Laboratory Standards Institute (CLSI) [12].

### 2.5. Screening of the *mecA* Gene in MDR CoNS

MDR CoNS, especially those found to be MR and cefoxitin-resistant, were further checked for the presence of the *mecA* gene, which is a standard molecular technique for MR confirmation according to the CLSI [13]. The detection of the *mecA* gene was carried out for all selected CoNS' isolates. DNA was extracted using a BioBee bacterial DNA extraction kit and used as a template in the PCR.

The 22-mer oligonucleotide primers were used as per a published report by Murakami et al. 1991 [14]. The classical *mecA* gene–targeting primers were used to amplify the target with approximately 533 base pairs of the gene, which encodes a low-affinity penicillin-binding protein (PBP2′), as described: Forward 5′ AAA ATC GAT GGT AAA GGT TGG C 3′ and reverse 5′ AGT TCT GCA GTA CCG GAT TTG C 3′. The PCR was performed using 2.5 μL of template DNA, 1 μL of forward and reverse primers, 8 μL of distilled water, and 12.5 μL of 2x Master Mix (Aura) in total 25 μL of reaction mixture. DNA amplification was carried out at 40 cycles of denaturation at 94°C for 30 s, annealing at 55°C for 30 s, and extension at 72°C for 1 min, with a final extension at 72°C for 5 min, by using an ABI thermal cycler. Amplification of the PCR product was checked on 2% agarose gel electrophoresis. Images were captured and analyzed by a gel document system (Bio-Rad).

### 2.6. *mecA* Gene Sequencing and Phylogeny

PCR–positive samples were subjected to Sanger sequencing, and the sequences obtained from the *mecA* gene were confirmed via BLASTn for homology, followed by phylogenetic analysis using MEGA 11 software once the top 5 homologs were used to construct the phylogram. In addition, another phylogram was constructed by aligning the *mecA* genes of UBEs and hospital settings CoNS to record the clades among sequences of the Vidarbha region targeted for the *mecA* gene.

### 2.7. SNP Mapping

Bioinformatics tools were used to record the prevalent mutations among the sequences of *mecA* genes. Multiple sequence alignments (MSAs) available in BioEdit software were used to carry out SNP analysis to record the aligned and unaligned regions. The unaligned regions among the sequenced *mecA* genes were carefully edited at the 5′ and 3′ end regions to analyze mutations among the sequences. The trimmed sequences were then saved in FASTA format. The sequences in the aligned format were subsequently used as input to DnaSP software, which has the capability to analyze DNA sequence polymorphisms. Among the aligned sequences, a total of nine sequences of the *mecA* gene belonging to the hospital and UBEs origin were obtained as follows: *S. saprophyticus* strain H26 (Acc no. PP466901), *S. cohnii* strain H31 (Acc no. PP466902), *S*. *haemolyticus* strain H42 (Acc no. PP466903), *S*. *saprophyticus* strain H47 (Acc no. PP466904), *S*. *haemolyticus* strain P7 (Acc no. PP466905), *S*. *haemolyticus* strain P9 (Acc no. PP466906), *S. warneri* strain P13 (Acc no. PP466907), *S*. *haemolyticus* strain P14 (Acc no. PP466908), and *S*. *cohnii* strain P7 (Acc no. PP466909). In addition to the tested species that were resistant to methicillin, one standard sequence has also been considered to confirm the occurrence of mutations among aligned sequences so that SNPs could be recorded within the standard sequence of the *S*. *aureus* subsp. *aureus* N315 *mecA* gene (Acc no. NG_047938.1). After realigning with multiple sequence alignments via BioEdit software, the resulting aligned sequences were fed into DnaSP software for SNP analysis.

### 2.8. MIC Via VITEK 2

Finally, all nine *mecA*-positive CoNS were further analyzed for MIC assay via automated VITEK 2 system using VITEK cards for 16 antibiotics, namely, benzylpenicillin, oxacillin, gentamicin, ciprofloxacin, levofloxacin, erythromycin, clindamycin, linezolid, daptomycin, teicoplanin, vancomycin, tetracycline, tigecycline, nitrofurantoin, rifampicin, and trimethoprim/sulfamethoxazole. In addition, cefoxitin screen and inducible clindamycin resistance were also checked for the presence of the marker ß-lactamase. The % of antibiotic susceptibility was checked for each CoNS isolate.

## 3. Results

### 3.1. Screening, Isolation, and Identification of *Staphylococcus* Species

200 different *Staphylococcus* isolates were recovered from the sample area (UBEs and hospital settings). Two distinct types of colonies were grown on MSA; pink colonies were identified as CoNS strains, while circular yellow colonies were identified as *S. aureus*. Further Gram staining and biochemical tests (catalase and coagulase tests) confirmed these isolates as *Staphylococcus* species.

### 3.2. Antibiotic Susceptibility Pattern and MDR Profiling of *Staphylococcus* Species

As per AST, 55 (27.50%) *Staphylococcus* isolates were found to be resistant to methicillin antibiotics, which were further tested to other antibiotics such as penicillin, oxacillin, and cefoxitin. As per the CLSI cut-off, out of 55 *Staphylococcus* isolates, 44 isolates were found to be MDR among the groups (UBEs and hospital settings). MDR *Staphylococcus* isolates were more prevalent in hospital settings (93.93%) than UBEs (59.07%) as shown in Table 1.

### 3.3. Identification and Characterization of Antibiotic-Resistant CoNS Via VITEK 2

A total of 44 MDR *staphylococcus* isolates were identified and characterized via the VITEK 2 automated system. Among them, 36 were identified as MDR–MRCoNS such as *S*. *saprophyticus*, *S*. *haemolyticus*, *S*. *warneri*, and *S*. *cohnii,* and the remaining 8 were *S*. *aureus* as recorded in Table 1 [15]. These 36 MDR–MRCoNS were checked for cefoxitin resistance and found that 19 were cefoxitin-resistant CoNS a shown in Table 1.

### 3.4. *mecA* Gene Presence

**Figure 1:**
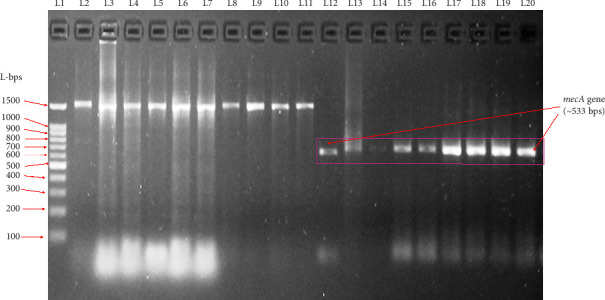
1.5% Agrose gel of the multidrug-resistant CoNS strains isolated from UBEs and the hospital settings. L 1: molecular marker. L2–11: *mecA-*negative CoNS. L12–L20: *mecA-*positive CoNS (∼533 bps band).

In the present study, a total of 19 MR and cefoxitin-resistant CoNS strains (9 belong to the UBEs and 10 belong to hospital settings) were screened for the presence of the *mecA* gene by conventional PCR.  Figure 1 shows that samples in L2–L11 were *mecA*-negative due to the absence of the ∼533 bps band; however, samples in L12–L20 were *mecA-*positive showing the amplification of the *mecA* gene (∼533 bps). L1 lane represents the 100 bps molecular ladder. These nine *mecA*-positive CoNS samples were identified and characterized as *S*. *haemolyticus* (*n* = 4), *S*. *saprophyticus* (*n* = 2), *S*. *warneri* (*n* = 1), and *S*. *cohnii* (*n* = 2) by VITEK 2.

### 3.5. Sanger Sequencing of Amplicon and Phylogeny

**Figure 2:**
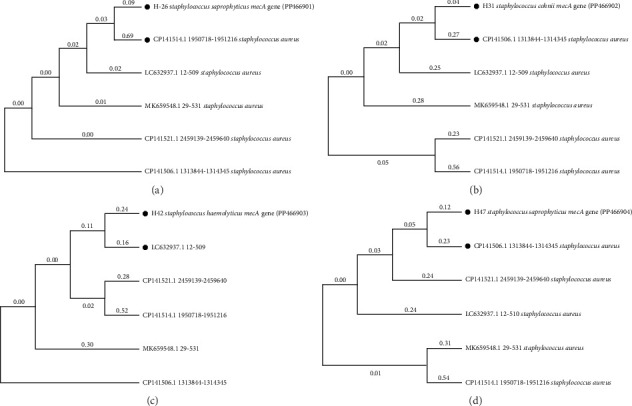
Phylograms of sequenced *mecA* gene–positive hospital MRCoNS individually matched with the best-scoring homologs: (a) H26 *Staphylococcus saprophyticus mecA* gene (PP466901), (b) H31 *Staphylococcus cohnii mecA* gene (PP466902), (c) H42 *Staphylococcus haemolyticus mecA* gene (PP466903), and (d) H47 *Staphylococcus saprophyticus mecA* gene (PP466904).

**Figure 3:**
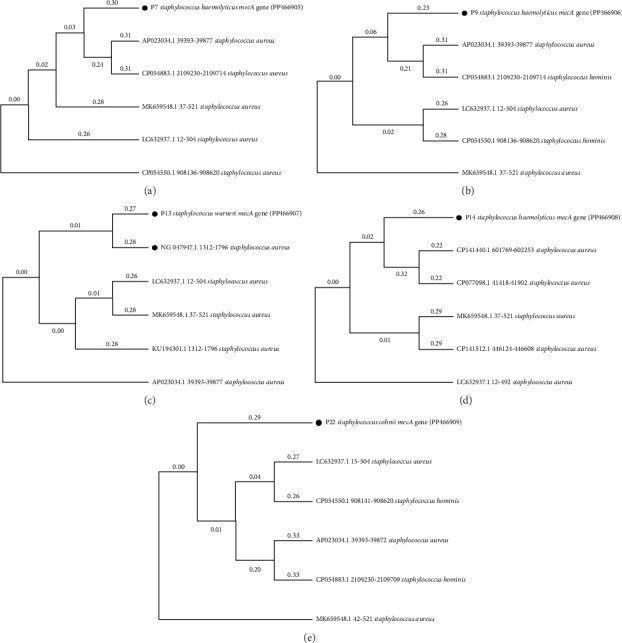
Phylograms of sequenced *mecA* gene–positive UBE MRCoNS individually matched with the best-scoring homologs: (a) P7 *Staphylococcus haemolyticus mecA* gene (PP466905), (b) P9 *Staphylococcus haemolyticus mecA* gene (PP466906), (c) P13 *Staphylococcus warneri mecA* gene (PP466907), (d) P14 *Staphylococcus haemolyticus mecA* gene (PP466908), and (e) P22 *Staphylococcus cohnii mecA* gene (PP466909).

Amplified products (*mecA* gene) of all nine *mecA*-positive samples (4 hospital CoNS and 5 UBEs' CoNS strains) were sequenced. Among them, the hospital strain H26 identified as *S*. *saprophyticus* was 526 bp in length, *S*. *cohnii* H31 was 520 bp in length, *S*. *haemolyticus* H42 was 520 bp in length, and *S*. *saprophyticus* H47 was 523 bp in length; similarly, the UBEs' strains identified as *S*. *haemolyticus* P7 was 516 bp in length, the *S*. *haemolyticus* P9 was 515 bp in length, *S*. *warneri* P13 was 520 bp in length, *S*. *haemolyticus* P14 was 519 bp in length, and the *S*. *cohnii* P22 was 516 bp in length. A phylogeny tree was prepared for all nine *mecA* gene sequences, which were individually matched with the best-scoring homologs; the five best-scoring homologs were identified and represented as phylograms in Figures 2 and 3.

**Figure 4:**
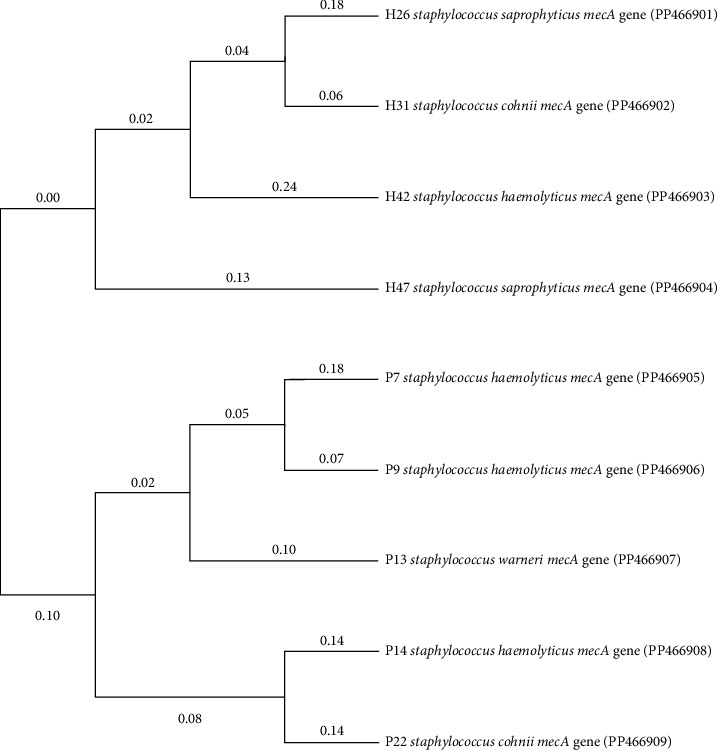
Phylogram of all nine sequenced *mecA* gene–positive UBEs and hospital MRCoNS.

In addition, all nine sequenced *mecA* genes of the UBEs and hospital MRCoNS aligned and phylogram developed (Figure 4), which showed that hospital and UBEs have two separate clades. Therefore, it distinctly indicates that certain unique gene sequence features separate them to be distributed among different environments (UBEs and hospital settings).

### 3.6. SNP Mapping

In the present study, a total of nine *mecA* gene sequences of UBE and hospital MRCoNS were successfully aligned for SNP analysis along with one standard sequence of *mecA* belonging to *S. aureus* (Accession no. NG_047938.1). The available nucleotide variants are reported as SNPs according to the standard sequence. All sequences were aligned with error correction using the BioEdit alignment tool, which served as inputs for the DNA sequence polymorphism software.

According to sequence alignment and SNP analysis, a total of 504 nucleotides were searched for mutations, with 466 sites aligned, excluding sites with gaps or missing data. Among these 466 nucleotides, 386 sequences were found to be invariable (monomorphic) out of the total 10 sequences. In addition, the total number of mutations recorded among the aligned sequences was variable (polymorphic), amounting to 80. This included 46 singleton variable sites and 34 parsimony information sites. Out of the singleton variable sites, 44 had two variants, and among the parsimony informative sites, 30 had two variants. There were also 2 singleton variable sites recorded with three variants, and among the parsimony informative sites, 4 had three variants, as shown in Table 2. A detailed comparative analysis of all the samples along with standard indicated the various SNPs and mutations in the *mecA* gene fragment (shown in Supporting table 1).

### 3.7. AST of *mecA* Gene–Positive MRCoNS Strains

Finally, the *mecA* gene–positive MRCoNS strains were tested for the AST via VITEK 2 against 16 antibiotics and the % of susceptibility is tabulated in Table 3. Cumulative % of susceptibility has shown (Table 3) that all the *mecA-*positive strains were 100% susceptible for gentamicin, ciprofloxacin, levofloxacin, tetracycline, tigecycline, nitrofurantoin, and trimethoprim/sulfamethoxazole. However, all strains were 100% resistant to benzylpenicillin and oxacillin drugs and approximately 90% resistant to vancomycin drugs. All isolates were also positive for ß-lactamase and cefoxitin screen, as shown in Table 3.

## 4. Discussion

In India, the healthcare industry is rapidly expanding to provide better health services and improve patient outcomes as discussed in the Ministry of Health and Family Welfare [16]. As per Sustainable Development Goals (SDGs) 2018, it is important to improve healthcare by achieving targeted SDGs through improvements in all directions [17]. According to WHO Health Statistics 2016, the incidence of microbial infections is on the rise in hospital settings, leading to mortality in many cases [18]. Earlier reports indicated that India's average life expectancy (68.3 years) is approximately 10 years shorter than that of the Maldives, which suggested that several shortcomings in healthcare sectors and their management have drawn attention [19]. Therefore, the Government of India started many healthcare programs with broader policy objectives to ensure translation in healthcare sectors [19]. According to surveillance studies, a recent increase in CoNS was reported as a common contaminant of nosocomial infections and was correlated with antibiotic resistance in India [20]. The present study has also shown that the presence of CoNS is very common and dominant in hospital settings and UBEs. According to Singh et al., in Indian tertiary care hospitals, CoNS were reported as the dominant species (*S*. *haemolyticus*, *S*. *epidermidis*, *S. hominis*, *S*. *cohnii* and *S*. *warneri)*. In addition, they have reported that most CoNS were MR (61.8% population SCC*mec* Type I–positive) [20]. Various other studies also reported similar profiles of CoNS and their drug resistance pattern in the hospital settings of India [21–23]. The Indian population resides in several countries around the globe, and comparatively, due to smaller land space, it easily shares the touch points by the number of people, which may lead to exposure and spreading of microbes especially CoNS in UBEs. Previous reports indicated that the prevalence of CoNS among academic institutions/universities and shrines of India, have increased which may be due to the exposure and spreading by the carrier's population [24–26]. In the present study, the prevalence of CoNS was found to be common on frequently touched surfaces of UBEs and hospital settings with MR features.

In this study, we reported that CoNS isolated from UBEs and hospital setting environments of the Vidarbha region of Maharashtra, India, were resistant to benzylpenicillin, oxacillin, and methicillin, in addition to the other antibiotics, which were similar to the previously published report [21] that highlighted the increased prevalence of MR *S. aureus* (MRSA) in India. Other studies also reported the MRCoNS from various zones of India, which were *mecA-*positive by molecular approach [21, 22, 27].

The present study reported thirty-six MDR–MRCoNS from hospital settings and UBEs were successfully identified at the species level by the automated VITEK 2 system, which were *S*. *haemolyticus*, *S*. *saprophyticus*, *S*. *warneri*, and *S*. *cohnii.* Among the 36 MDR–MRCoNS, 19 were cefoxitin-resistant CoNS, which were analyzed for the presence of the *mecA* gene at the molecular level. Among the 19 cefoxitin-resistant CoNS, 9 (47.36%) were found to be *mecA* gene–positive by conventional PCR, which confirmed the presence of MR and cefoxitin resistance linkages with the *mecA* gene. Therefore, MR and cefoxitin resistance were an average indicator of *mecA* gene–mediated resistance, which suggests that certain other mechanisms could also have contributed to MR and cefoxitin resistance in CoNS [8]. Earlier published reports indicate that 45%–68% of CoNS showed cefoxitin resistance due to the harboring of the *mecA* gene and the rest 32%–55% of CoNS showed cefoxitin resistance due to some other existing mechanisms rather than *mecA* gene [8, 23, 28]. Recently Felgate et al. reported that 50% of cefoxitin-resistant CoNS isolates did not contain *mec* homolog [29].

In this study, MDR was more prevalent among MRCoNS strains isolated from hospital settings (83.87%) than among those isolated from UBEs (76.92%), with a significant presence of *mecA* genes. Similarly, Ahmad et al. reported MRCoNS isolates from hospital personnel and environments, which showed antibiotic resistance [30]. The current study also revealed a distinct segregation of *mecA* gene lineages between CoNS species from UBE and hospital settings through sequencing and phylogenetic analysis, which suggests that a unique lineage of strains spreads across different environments. Similar observations were also reported by Sharma, suggesting the horizontal transmission of *mecA* in MRSA; however, Balachander and Alexander linked MR and virulence in CoNS through phylogenetic analysis of efflux proteins within *mecA*-positive strains [31, 32].

Furthermore, SNP and mutational analysis of the *mecA* gene in MRCoNS was found to be informative, with 80 variables (polymorphic) among the 466 nucleotides studied. Salehi et al. reported the association between SNPs and antibiotic resistance in MR staphylococci, and 10% of clinical specimens were found to be SNP–positive in the *mecA* gene. They utilized the chi-square test to link the associations between SNPs in the *mecA* gene and resistance to cefoxitin, oxacillin, and erythromycin in clinical isolates [33]. Fluit et al. suggested that MRCoNS induces the expression of the low-affinity PBP, which is encoded by the *mecA* gene, which was carried by the staphylococcal cassette chromosome (SCC). A study suggests that the *mecA* and *ccrB* gene sequences are identical in many cases of CoNS, which are *S*. *aureus*, because of the frequent horizontal transfer of SCC*mec* [34].

The MIC assay in our study confirmed that all MRCoNS were resistant to high concentrations of benzylpenicillin and oxacillin, and approximately, 50% of the strains were resistant to linezolid, indicating an increasing resistance profile toward newer generation drugs and heightened virulence. The assay highlighted that the most sensitive species, *S*. *warneri* was resistant to only benzylpenicillin and oxacillin, as compared to *S*. *cohnii*, *S*. *haemolyticus*, and *S*. *saprophyticus,* which were resistant to multiple drugs. These findings are consistent with Oommen, Appalaraju, and Jinsha, who reported high- and low-level mupirocin resistance in MRCoNS [35]. Despite the low virulence of *S. haemolyticus*, as noted by Manoharan, Sistla, and Ray, it remains a frequent infection agent due to its resistance to high dosages of cefoxitin, erythromycin, cotrimoxazole, clindamycin, and, recently, linezolid, although our study did not record resistance to linezolid (MIC assay range 4.3 ± 4.19), indicating borderline sensitivity that requires monitoring [36]. Singh et al. also highlighted increasing antimicrobial resistance in CoNS in Indian tertiary care hospitals [37]. Our study underscores the rising incidence of CoNS' persistence and drug resistance in both UBEs and hospital environments, indicating an urgent need for ongoing surveillance and management strategies.

## 5. Conclusion

In developing countries such as India, where the larger population lives together and shares a small portion of where they serve, large areas of hospital environments and UBEs are being contacted by virulent MRCoNS strains having MDR profiles. As investigated in Vidarbha, where MRCoNS strains are MDR, they have genome positivity for the *mecA* gene, and many mutations are linked with every species of staphylococci. The spread of antibiotic resistance is very common in both hospital environments and UBEs; therefore, the current study suggested that a surveillance program is needed in the Vidarbha region of Maharashtra, India, for the assessment of the prevalence of CoNS and its improved management in the environment for future reduction of the contact infection.

## Figures and Tables

**Table 1:** Methicillin and multidrug resistance profiling of *Staphylococcus* species, and cefoxitin-resistant CoNS' screening and characterization by the presence of the *mecA* gene.

S. no	Sites	Number of *staphylococcus* isolates separated from collected samples *n* (%)	Methicillin-susceptible *Staphylococcus* isolates *n* (%)	Methicillin-resistant *Staphylococcus* isolates *n* (%)	Multidrug-resistant–methicillin-resistant *Staphylococcus* isolates *n* (%)	Identification of MDR *S. aureus* via VITEK 2 *n* (%)	Identification of MDR CoNS via VITEK 2 *n* (%)	Cefoxitin-resistant CoNS *n* (%)	*mecA-*positive CoNS via PCR *n* (%)	*mecA-*negative CoNS *n* (%)
1	Urban built environments (UBEs)	100	78/100 (78%)	22/100 (22%)	13/22 (59.09%)	3/13 (23.07%)	10/13 (76.92%)	9/10 (90%)	5/9 (55.55%)	4/9 (44.44%)
2	Hospital settings	100	67/100 (67%)	33/100 (33%)	31/33 (93.93%)	5/31 (16.12%)	26/31 (83.87%)	10/26 (38.46%)	4/10 (40%)	6/10 (60%)
3	Grand total	200	145/200 (72.50%)	55/200 (27.50)	44/55 (80%)	8/44 (18.18%)	36/44 (81.81%)	19/36 (52.77%)	9/19 (47.36%)	10/19 (52.63)

*Note:* In CoNS, we have identified four species, namely, *S. saprophyticus*, S. *haemolyticus*, *S. warneri*, and *S. cohnii*.

**Table 2:** Parsimony informative sites (PISs) and other sites among the four identified species in nine samples (4 hospital sites and 5 UBE sites).

Species	Sequences/sites analyzed	MS	PS	SV	PIS	SV2V	PIS2V	SV3V	PIS3V
*S. haemolyticus*	4/504 bp	425	51	50	1	50	1	0	0
*S. saprophyticus*	2/504 bp	486	4	4	0	4	0	0	0
*S. cohnii*	2/504 bp	432	47	47	0	47	0	0	0
*S. warneri*	1/504 bp	488	0	0	0	0	0	0	0
All aligned	9/504 bp	396	72	39	33	37	31	2	2
All aligned with RefSeq (Accession no. NG_047938.1)	10/504 bp	386	80	46	34	44	30	2	4

*Note:* SV, singleton variable sites.

Abbreviations: MS, monomorphic sites; PISs, parsimony informative sites; PIS2V, parsimony informative sites with two variants; PIS3V, parsimony informative sites with three variants; PS, polymorphic sites; SV2V, singleton variable sites with two variants; SV3V, singleton variable with three variants.

**Table 3:** MIC data of *mecA-*positive CoNS' samples at the species level via an automated VITEK 2 system.

Antibiotics	*S. haemolyticus* (n = 04)		*S. saprophyticus* (*n = *02)		*S. warneri* (*n = *01)		*S. cohnii* (*n = *02)		Cumulative % of S
	MIC (μg/mL)	Mean ± SD (% R/S)	MIC (μg/mL)	Mean ± SD (% R/S)	MIC (μg/mL)	Mean ± SD (% R/S)	MIC (μg/mL)	Mean ± SD (% R/S)	
Benzylpenicillin	0.03–0.5	0.38 ± 0.23 (100% R)	0.5	0.5 ± 0.00 (100% R)	0.5	0.5 ± 0.00 (100% R)	0.5–0.25	0.375 ± 0.17 (100% R)	0
Oxacillin	4	4 ± 0.00 (100% R)	4	4 ± 0.00 (100% R)	4	4 ± 0.00 (100% R)	4	4 ± 0.00 (100% R)	0
Gentamicin	0.5	0.5 ± 0.00 (100% S)	0.5–1	0.75 ± 0.35 (100% S)	0.5	0.5 ± 0.00 (100% S)	0.5	0.5 ± 0.00 (100% S)	100
Ciprofloxacin	0.5	0.5 ± 0.00 (100% S)	0.5	0.5 ± 0.00 (100% S)	0.5	0.5 ± 0.00 (100% S)	0.5	0.5 ± 0.00 (100% S)	100
Levofloxacin	0.12–0.25	0.18 ± 0.07 (100% S)	0.12–0.5	0.31 ± 0.26 (100% S)	0.12	0.12 ± 0.00 (100% S)	0.12–0.25	0.185 ± 0.09 (100% S)	100
Erythromycin	0.25–8	6.06 ± 3.8 (75% R)	8	8 ± 0.00 (100% R)	0.25	0.25 ± 0.00 (100% S)	8	8 ± 0.00 (100% R)	22.22
Clindamycin	0.12–4	3.03 ± 1.94 (75% R)	4	4 ± 0.00 (100% R)	0.25	0.25 ± 0.00 (100% S)	0.5–4	2.25 ± 2.47 (100% R)	22.22
Linezolid	0.5–8	4.3 ± 4.19 (50% R)	8	8 ± 0.00 (100% R)	0.5	0.5 ± 0.00 (100% S)	01–04	2.5 ± 2.12 (100% S)	55.55
Daptomycin	8	8 ±0 .0.00 (100% R)	8	8 ± 0.00 (100% R)	2	2 ± 0.00 (100% S)	0.12–4	2.06 ± 2.74 (100% S)	33.33
Teicoplanin	0.5–32	24.1 ± 15.7 (75% R)	32	32 ± 0.00 (100% R)	0.5	0.5 ± 0.00 (100% S)	02–32	17 ± 21.21 (50% R)	33.33
Vancomycin	32	32 ± 0.00 (100% R)	32	32 ± 0.00 (100% R)	0.5	0.5 ± 0.00 (100% S)	32	32 ± 0.00 (100% R)	11.11
Tetracycline	01–02	1.25 ± 0.5 (100% S)	01–02	1.5 ± 0.7 (100% S)	1	1 ± 0.00 (100% S)	1	1 ± 0.00 (100% S)	100
Tigecycline	0.12	0.12 ± 0.00 (100% S)	0.12–0.25	0.18 ± 0.09 (100% S)	0.12	0.12 ± 0.00 (100% S)	0.12–0.25	0.185 ± 0.09 (100% S)	100
Nitrofurantoin	16–32	24 ± 9.23 (100% S)	16	16 ± 0.00 (100% S)	16	16 ± 0.00 (100% S)	16–32	24 ± 11.31 (100% S)	100
Rifampicin	0.03–4	2.01 ± 2.29 (50% R)	4	4 ± 0.00 (100% R)	1	1 ± 0.00 (100% S)	4	4 ± 0.00 (100% R)	33.33
Trimethoprim/sulfamethoxazole	10	10 ± 0.00 (100% S)	10	10 ± 0.00 (100% S)	10	10 ± 0.00 (100% S)	10	10 ± 0.00 (100% S)	100
Beta-lactamase	POS		POS		POS		POS		
Cefoxitin screen	POS		POS		POS		POS		
Inducible clindamycin resistance	NEG		NEG		NEG		NEG		

## Data Availability

The data used to support the findings of this study are included within the article.

## Supporting Information

Supporting Table S1: Detailed comparative analysis of all samples, identifying SNPs and mutations in the *mecA* gene fragment. The table includes variable and parsimony informative sites categorized by their occurrence, highlighting specific mutations across strains referenced to the standard NG_047938.1.

Supporting Table 1: Detailed comparative SNP analysis of all the *mecA-*positive strains compared to RefSeq (Accession no. NG_047938.1).
